# Asymmetrical than symmetrical cerebral arterial bifurcations are more vulnerable to aneurysm presence

**DOI:** 10.1038/s41598-019-53715-z

**Published:** 2019-11-20

**Authors:** Xue-Jing Zhang, Wei-Li Hao, Dong-Hai Zhang, Bu-Lang Gao

**Affiliations:** 1 Department of Medical Research, Shijiazhuang First Hospital, Shijiazhuang, China; 2 Henan Balance Medial Laboratory, Henan Balance Medical Company, Zhengzhou, China

**Keywords:** Stroke, Outcomes research

## Abstract

In order to investigate the role of lateral angle ratio (LA ratio) and daughter artery ratio (DA ratio) for predicting aneurysmal presence in main cerebral arterial bifurcations, three-dimensional cerebral angiographic data of major cerebral artery bifurcations were used for measurement of artery diameters and bifurcation angles including 115 middle cerebral arteries (MCAs), 59 basilar arteries (BAs), 35 internal carotid arteries (ICAs) and 115 anterior cerebral arteries (ACAs) with bifurcation aneurysms and control subjects of 1921 bifurcations with no aneurysms. The LA ratio (larger lateral angle/smaller lateral angle) and DA ratio (larger branch diameter/smaller branch diameter) were calculated, and ROC curve analysis of LA and DA ratios between normal and aneurysmal cases was performed. The LA and DA ratios of MCA bifurcations and the LA ratios of BA and ICA bifurcations with aneurysms were all significantly larger than normal bifurcations (*P* < 0.05), and the DA ratio of ACA bifurcations with aneurysms was significantly smaller than normal cases (*P* < 0.01). Moreover, the LA ratio or DA ratio between the normal and aneurysm cases in MCA, BA and ACA bifurcations demonstrated significant differences by ROC analysis (*P* < 0.01) except in the ICA bifurcations. No significant difference was observed (*P* > 0.05) between ruptured and unruptured aneurysms in MCA, BA, ICA and ACA bifurcations. In summary, normal MCA, BA and ICA bifurcations show symmetrical morphology in the lateral angles and daughter branches, whereas aneurysmal bifurcations show asymmetrical morphology. Normal ACA bifurcations have asymmetrical bilateral daughter branches while symmetrical branches are associated with ACA bifurcation aneurysm presence.

## Introduction

Subarachnoid hemorrhage (SAH) is associated with high mortality, disability rates, and socioeconomic cost^1,2^, and cerebral aneurysm rupture is the cause of 85% of SAH^3^. Cerebral aneurysms occur in approximately 3% of the population^4^. Although the rupture rate is only 1% in all aneurysms^5^, the consequences are devastating. Thus, identification of bifurcations with a high risk of aneurysm presence and rupture would help surgical management and possible prevention. With fast advancement of noninvasive neuroimaging technologies, including digital subtraction angiography (DSA) and magnetic resonance angiography, arterial bifurcation geometry examination has become feasible. Significant differences in morphological factors^6,7^ have been observed between normal and aneurysmal MCA bifurcations, and between ruptured and unruptured aneurysmal bifurcations of MCAs, inferring that morphological parameters could help risk assessment of aneurysm presence and rupture.

Our previous studies^8,9^ showed that the anterior cerebral artery (ACA) and basilar artery (BA) bifurcations with aneurysms were associated with wider bifurcation angles and narrower bilateral angles and that most aneurysms deviated to the smaller lateral angle and smaller daughter branch. We thus hypothesized that asymmetrical bifurcation geometry in two lateral angles and two daughter branches was more vulnerable to aneurysm presence. It has been reported that normal MCA bifurcations have nearly symmetric structure, whereas aneurysmal MCA bifurcations present with asymmetrical anatomy^10^. Moreover, a higher AP ratio (the maximum dimension of the dome/aneurysm neck) and a smaller DA ratio (larger branch diameter/smaller branch diameter) were associated with MCA aneurysm rupture^11^. Currently, no studies have been performed to investigate lateral angles (LA) ratio and DA ratio in relation to aneurysm presence and rupture at major cerebral arterial bifurcations using a large amount of specific three-dimensional imaging data of patients with and without cerebral aneurysms. Consequently in this paper, we focused on analyzing the asymmetrical or symmetrical structure of major arterial bifurcations of MCA, BA, ICA and ACA with and without aneurysms. The purpose of this study was to determine whether the DA and LA ratio could be useful geometric indexes to distinguish aneurysmal from nonaneurysmal bifurcations and unruptured from ruptured aneurysms. This study about the symmetrical or asymmetrical bifurcation structure may provide clues for a link between morphological elements and aneurysm presence and rupture.

## Materials and Methods

**Figure 1:**
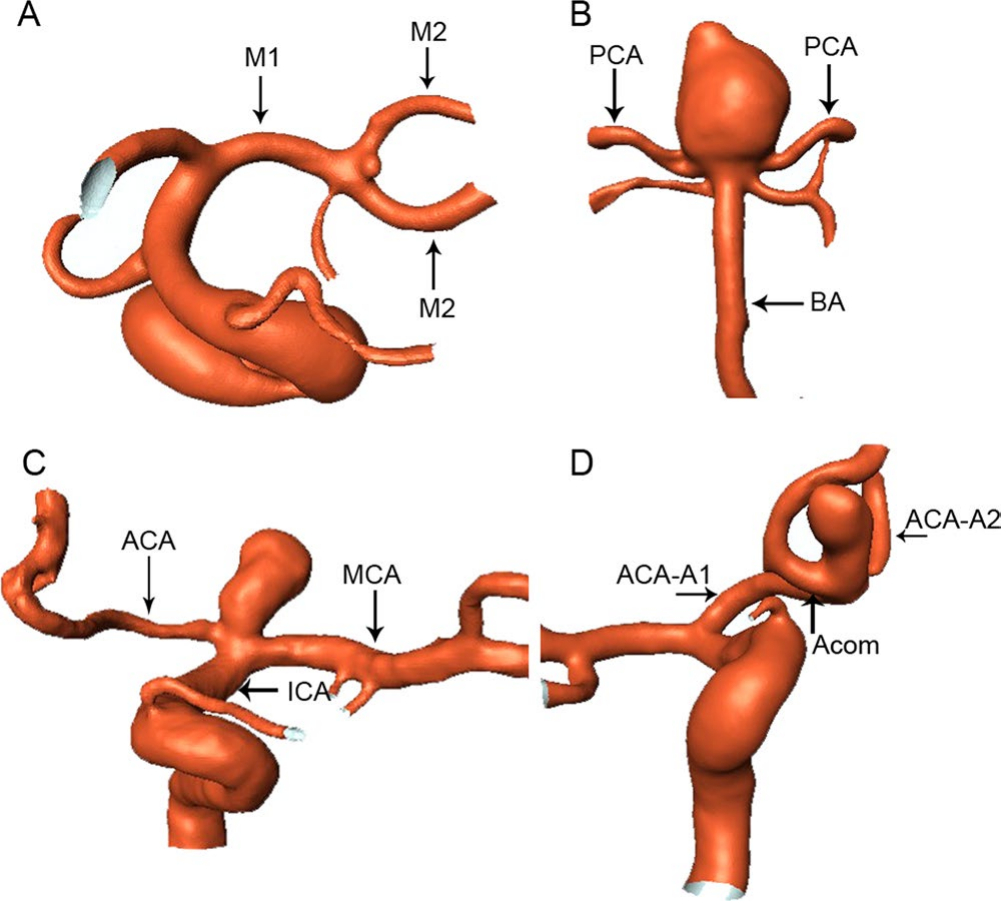
Aneurysms are shown at the bifurcations of the middle cerebral artery (MCA), basilar artery (BA), internal carotid artery (ICA) and anterior cerebral artery (ACA). (**A**) MCA bifurcation aneurysm. M1 and M2 represent M1 and M2 segments of middle cerebral artery, respectively. (**B**) BA bifurcation aneurysm. PCA represents posterior cerebral artery. (**C**) ICA bifurcation aneurysm. (**D**) ACA bifurcation aneurysm. ACA-A1, ACA-A2 and Acom represent the A1 and A2 segments of ACA and anterior communicating artery, respectively.

Between March 2004 and February 2015, consecutively registered patients who had three-dimensional digital subtraction angiography (DSA) in our hospital were reviewed in this study. All DSA volumes in the DICOM format showing a clear view of the major arterial bifurcations were included, and those with unclear imaging data were excluded. Computational fluid dynamic (CFD) analysis was performed in 324 major cerebral arterial bifurcations with cerebral aneurysms, including MCA (n = 115), BA (n = 59), ICA (n = 35) and ACA (n = 115) bifurcations based on these three-dimensional DSA data (Fig. 1). The symptoms of these patients included SAH, headache, confusion, face numbness, double vision and nonspecific neurological symptoms. Control subjects with no intracranial arterial stenosis or cerebral aneurysms, who had digital subtraction angiography for suspected cerebrovascular disease, were also recruited for major cerebral arterial bifurcations, including 684 MCA, 136 BA, 547 ICA and 439 ACA bifurcations, for comparison with the aneurysmal group (Table 1). Data on patients’ age, gender, symptoms and aneurysm status were collected from a prospectively maintained database. There was no statistically (*P* > 0.05) significant difference in the mean age or gender percentage between the aneurysmal and the control groups. This study was approved by the ethics committee of Shijiazhuang First Hospital (IRB approval number: IRB-2016001), and informed consent was obtained from all participants and/or their legal guardians. All methods were performed in accordance with the relevant guidelines and regulations.

**Table 1:** Baseline characteristics of patients.

	MCA bifurcations (N = 799)	BA bifurcations (N = 195)	ICA bifurcations (N = 582)	ACA bifurcations (N = 554)
**Gender**				
Female	558	110	403	335
Male	241	85	179	219
**Mean age in yrs** **(range)**	55.3 ± 13.9 (11–92)	53.0 ± 14.5 (18–82)	55.4 ± 13.9 (11–92)	52.6 ± 14.5 (11–92)
**Bifurcations features**				
Normal	684	136	547	439
With aneurysms	115	59	35	115
**Aneurysms features**				
Ruptured	8	5	2	15
Unruptured	107	54	33	100

Note: Data are shown as mean ± SD; MCA, middle cerebral artery; BA, basilar artery; ICA, internal carotid artery; ACA, anterior cerebral artery.

**Figure 2:**
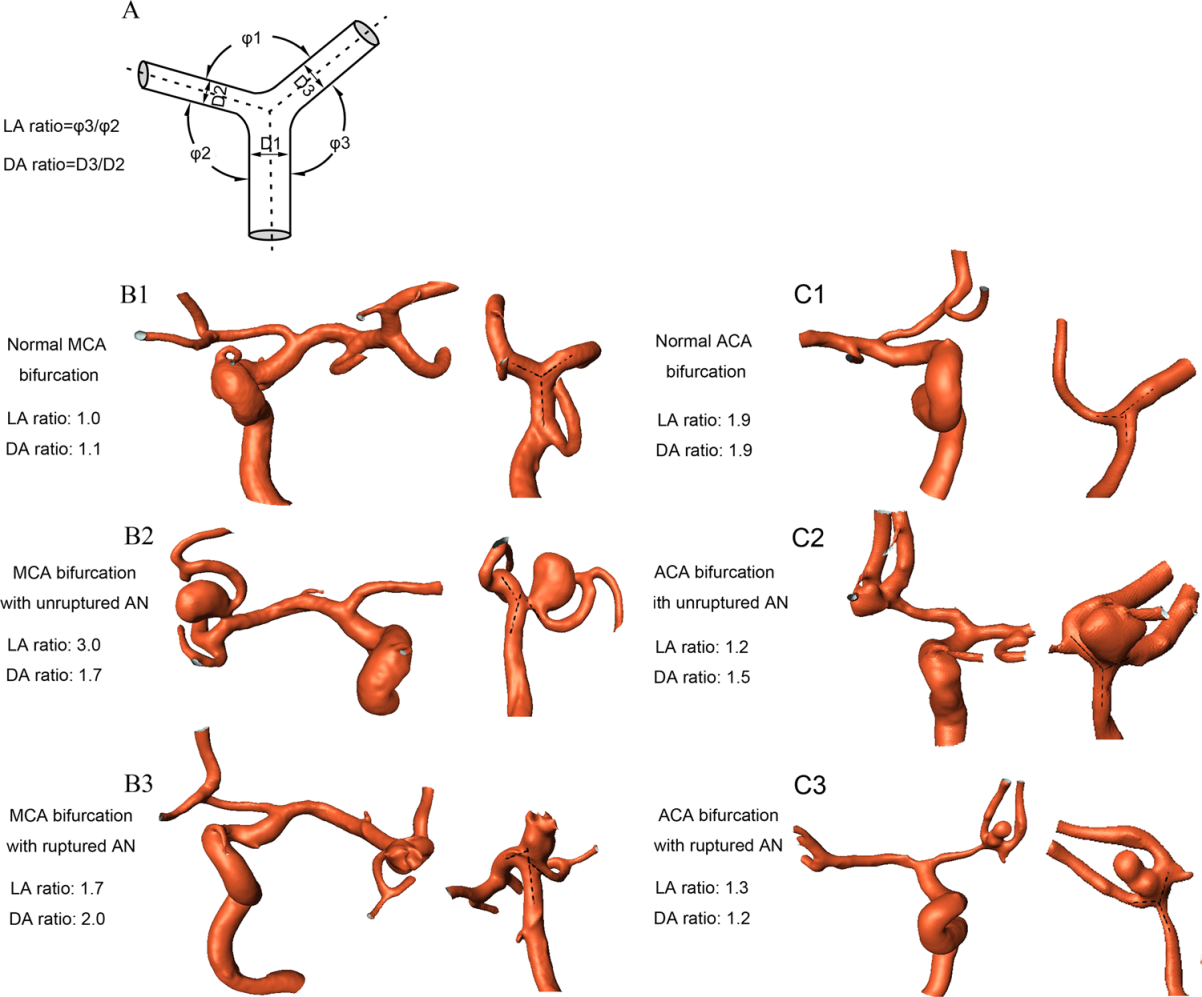
(**A**) Schematic drawing shows the definition of LA ratio and DA ratio. Larger lateral angle/smaller lateral angle was named as the LA ratio. The larger branch diameter/smaller branch diameter was termed the DA ratio. φ1, the bifurcation angle formed between bilateral daughter branches; φ2, smaller lateral angle; φ3, larger lateral angle; D1, diameter of parent vessel; D2 and D3, smaller and larger diameter of daughter branches, respectively. (**B1–3**) Normal MCA bifurcation (**B1**), MCA bifurcation with unruptured (**B2**) and ruptured (**B3**) aneurysm. (**C1**–**3**) Normal ACA bifurcation (**C1)**, ACA bifurcation with unruptured (**C2**) and ruptured (**C3**) aneurysm. AN, aneurysm.

Three-dimensional rotational angiography data in the DICOM format were reconstructed and transferred for surface rendering by using the Amira software (version 5.2.2, Visage Imaging, San Diego, California, USA). The angles formed between bilateral daughter branches (φ1) and bilateral angles formed between daughter and parent vessels (the smaller angle defined as φ2 and the larger one as φ3) were measured. As we reported previously^8,9^, angle φ1 was measured by use of 3 dots after the central point was placed at the tip of the bifurcation in line with the central axis of the parent artery, and the other 2 dots marked the central axis of the proximal bilateral daughter branches. Angles φ2 and φ3 were evaluated in a similar manner (Fig. 2A). The LA ratio was termed as φ3/φ2.

The diameter of parent and bilateral daughter vessels were measured in a similar way to the approach used by Ingebrigtsen *et al*.^12^ and us in the previous studies^8,9^. The diameter of the parent vessel was termed D1, the smaller daughter branch was defined as D2, and the larger daughter one as D3 (Fig. 2). D3/D2 was named as the DA ratio.

### Statistical analysis

All data presented in present study was based on untreated vessels from coiling/stenting and were presented as mean ± standard deviation. The JMP 10.01.2 statistical software (SAS Institute, Cary, NC, USA) was used for statistical analysis. The *t*-test was used to compare the morphological parameter differences between normal and aneurysmal cases. *P* value < 0.05 was considered to be statistically significant.

Receiver operating characteristics (ROC) curve analyses of the LA and DA ratios quantified between normal and aneurysmal cases by the area under the curve (AUC) index were used to determine the optimal discriminating thresholds for aneurysm presence and the LA and DA ratios between ruptured and unruptured cases for aneurysm rupture diagnosis.

## Results

Data from a total of 799 MCA, 195 BA, 582 ICA and 554 ACA bifurcations were available for analysis. The population subgroups of these major cerebral arterial bifurcations were control subjects, all aneurysmal bifurcations, ruptured and unruptured cases. The results of LA and DA ratios analysis and ROC analysis for evaluating cerebral aneurysm presence and rupture were as follows.

### LA and DA ratios of MCA bifurcations

In MCA bifurcations, the two lateral angles were significantly smaller in aneurysmal bifurcations than in normal bifurcations (*P* < 0.05), whereas angle φ1 was significantly greater in aneurysmal than in normal bifurcations (*P* < 0.001) (Table 2).

**Table 2:** Bifurcation angles and branch diameter in MCA, BA, ICA, ACA bifurcations.

		Normal	Total AN	Unrup AN	Rup AN
MCA bifurcations	φ1 (°)	102.8 ± 24.6	152.4 ± 35.0^***^	152.2 ± 34.7^***^	154.9 ± 43.6^***^
	φ2 (°)	111.3 ± 19.3	76.6 ± 22.9^***^	76.5 ± 23.2^***^	79.6 ± 19.5^***^
	φ3 (°)	138.2 ± 14.8	122.2 ± 24.1^**^	122.0 ± 24.3^**^	124.7 ± 23.7^*^
	D1(mm)	2.8 ± 1.0	2.5 ± 0.8	2.5 ± 0.8	2.1 ± 0.1
	D2 (mm)	1.9 ± 0.7	1.6 ± 0.6	1.6 ± 0.6	1.7 ± 0.9
	D3 (mm)	2.5 ± 0.9	2.4 ± 0.9	2.4 ± 0.8	2.9 ± 1.6
BA bifurcations	φ1 (°)	106.5 ± 19.7	140.8 ± 13.8^***^	140.4 ± 13.6^***^	144.9 ± 17.5^***^
	φ2 (°)	115.5 ± 15.2	91.5 ± 15.6^***^	91.6 ± 15.4^***^	90.3 ± 20.5^***^
	φ3 (°)	133.3 ± 11.6	116.6 ± 15.8^***^	116.4 ± 16.1^***^	119.3 ± 13.6^**^
	D1(mm)	4.8 ± 1.9	4.6 ± 2.0	4.5 ± 2.0	5.8 ± 2.8
	D2 (mm)	3.0 ± 1.3	2.8 ± 1.5	2.8 ± 1.6	3.1 ± 1.1
	D3 (mm)	3.7 ± 1.5	3.5 ± 1.9	3.4 ± 1.9	4.3 ± 2.2
ICA bifurcations	φ1 (°)	134.6 ± 45.8	140.2 ± 24.9	139.0 ± 24.3	173.3 ± 8.2
	φ2 (°)	76.4 ± 17.3	65.5 ± 17.9	65.5 ± 18.5	55.9 ± 3.1^*^
	φ3 (°)	138.0 ± 12.2	134.5 ± 15.0	135.3 ± 14.9	122.3 ± 6.2^*^
	D1(mm)	4.3 ± 2.6	4.2 ± 1.8	4.0 ± 1.6	8.2 ± 0.9^***^
	D2 (mm)	2.5 ± 1.1	2.7 ± 1.3	2.5 ± 1.0	5.2 ± 0.2^***^
	D3 (mm)	3.2 ± 1.4	3.4 ± 1.5	3.2 ± 1.3	6.7 ± 0.4^***^
ACA bifurcations	φ1 (°)	106.5 ± 14.6	133.8 ± 18.5^***^	133.3 ± 18.1^***^	136.9 ± 21.3^***^
	φ2 (°)	103.4 ± 15.6	94.0 ± 13.2^**^	94.9 ± 13.2^**^	87.9 ± 16.7^***^
	φ3 (°)	126.8 ± 13.6	114.0 ± 13.3^***^	114.0 ± 13.4^***^	114.1 ± 13.5^***^
	D1(mm)	4.1 ± 1.2	2.5 ± 1.0^***^	2.5 ± 0.9^***^	2.5 ± 1.3^***^
	D2 (mm)	2.5 ± 1.0	2.1 ± 0.9	2.0 ± 0.8^*^	2.4 ± 1.3
	D3 (mm)	4.1 ± 1.3	2.7 ± 0.9^***^	2.7 ± 0.9^***^	2.9 ± 1.3^***^

Note: Data are shown as mean ± SD. MCA, BA, ICA and ACA indicate middle cerebral artery, basilar artery, internal carotid artery and anterior cerebral artery, respectively. AN, aneurysms; φ1, the bifurcation angle formed between bilateral daughter branches; φ2, smaller lateral angle; φ3, larger lateral angle. D1, D2 and D3, diameter of parent vessel, smaller and larger daughter branches, respectively. ^*^P < 0.05, ^**^P < 0.01 and ^***^P < 0.001 compared with normal control.

**Figure 3:**
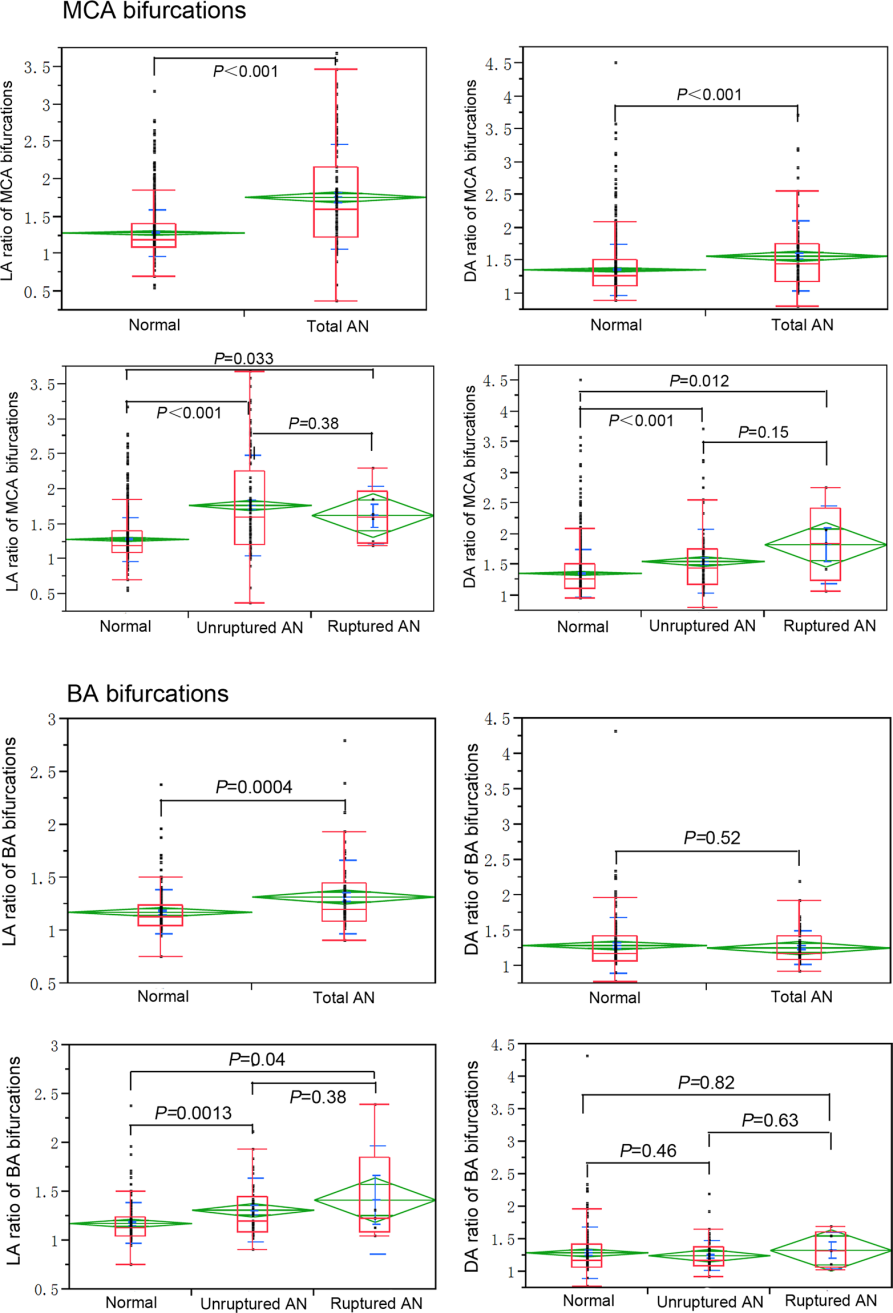
Comparison of LA and DA ratios in the middle cerebral artery (MCA) and basilar artery (BA) bifurcations between non-aneurysmal, unruptured and ruptured aneurysmal cases. Normal, control subjects without aneurysms; AN, aneurysms.

The LA ratio was significantly (*P* < 0.001) greater in aneurysmal MCA bifurcations (1.8 ± 0.7) than in normal MCA cases (1.3 ± 0.3). Furthermore, the LA ratio was also significantly greater in unruptured (*P* < 0.001) and ruptured (*P* = 0.033) aneurysmal cases than in normal control subjects (Fig. 3). The DA ratio of normal, total aneurysmal cases, unruptured and ruptured aneurysmal cases was 1.4 ± 0.4, 1.6 ± 0.5, 1.6 ± 0.5 and 1.8 ± 0.6, respectively, with a significantly smaller DA ratio in the normal control subjects than in the rest (*P* < 0.001, *P* < 0.001 and *P* = 0.012, respectively) (Fig. 3). No significant differences in the LA and DA ratios between ruptured and unruptured cases were observed (*P* > 0.05).

Both the LA and DA ratios between normal MCA bifurcations and those with aneurysms (including the two categories of total and unruptured aneurysmal cases) demonstrated significant differences by ROC analysis (*P* < 0.001), and the AUC index was 0.74 in the LA ratio between normal and aneurysmal cases, 0.73 in the LA ratio between normal and unruptured aneurysmal cases, 0.64 in the DA ratio between normal and aneurysmal cases and 0.63 in the DA ratio between normal and unruptured aneurysms cases (Table 3). Although there was a significant difference in the DA ratio between normal control subjects and ruptured aneurysmal cases in student’s t test(*P* < 0.05), no significant difference was observed in ROC analysis (*P* = 0.06) (Table 3).

**Table 3:** ROC curve analysis of LA and DA ratios for predicting aneurysm presence and rupture status.

	LA ratio	*P*	AUC index	Cut point	DA ratio	*P*	AUC index	Cut point
**MCA bifurcations**								
Normal	1.3 ± 0.3	<0.001	0.74	1.55	1.4 ± 0.4	<0.001	0.64	1.38
Total AN	1.8 ± 0.7				1.6 ± 0.5			
Normal	1.3 ± 0.3	=0.006	0.86	1.57	1.4 ± 0.4	=0.06	0.74	1.84
Rup AN	1.6 ± 0.4				1.8 ± 0.6			
Normal	1.3 ± 0.3	<0.001	0.73	1.55	1.4 ± 0.4	<0.001	0.63	1.38
Unrup AN	1.8 ± 0.7				1.6 ± 0.5			
Rup AN	1.6 ± 0.4	=0.74	0.55	1.55	1.8 ± 0.6	=0.30	0.64	1.82
Unrup AN	1.8 ± 0.7				1.6 ± 0.5			
**BA bifurcations**								
Normal	1.2 ± 0.2	=0.001	0.56	1.30	1.3 ± 0.4	=0.68	0.52	1.10
Total AN	1.3 ± 0.3				1.3 ± 0.2			
Normal	1.2 ± 0.2	=0.19	0.67	1.12	1.3 ± 0.4	=0.60	0.57	1.30
Rup AN	1.4 ± 0.6				1.3 ± 0.3			
Normal	1.2 ± 0.2	=0.002	0.64	1.31	1.3 ± 0.4	=0.77	0.51	1.08
Unrup AN	1.3 ± 0.3				1.2 ± 0.2			
Rup AN	1.4 ± 0.6	=0.94	0.51	2.25	1.3 ± 0.3	=0.57	0.58	1.31
Unrup AN	1.3 ± 0.3				1.2 ± 0.2			
**ICA bifurcations**								
Normal	1.9 ± 0.6	=0.10	0.60	1.98	1.3 ± 0.3	=0.83	0.51	1.09
Total AN	2.2 ± 0.7				1.3 ± 0.2			
Normal	1.9 ± 0.6	=0.25	0.74	2.17	1.3 ± 0.3	=0.87	0.53	1.26
Rup AN	2.2 ± 0.01				1.3 ± 0.02			
Normal	1.9 ± 0.6	=0.16	0.58	2.0	1.3 ± 0.3	=0.86	0.51	1.09
Unrup AN	2.2 ± 0.7				1.3 ± 0.2			
Rup AN	2.2 ± 0.01	=0.52	0.64	2.14	1.3 ± 0.02	=0.85	0.50	1.25
Unrup AN	2.2 ± 0.7				1.3 ± 0.2			
**ACA bifurcations**								
Normal	1.3 ± 0.3	=0.75	0.51	1.33	1.8 ± 0.8	<0.001	0.70	1.50
Total AN	1.2 ± 0.2				1.4 ± 0.4			
Normal	1.3 ± 0.3	=0.11	0.38	1.36	1.8 ± 0.8	=0.001	0.75	1.30
Rup AN	1.3 ± 0.3				1.3 ± 0.4			
Normal	1.3 ± 0.3	=0.36	0.53	1.23	1.8 ± 0.8	<0.001	0.70	1.50
Unrup AN	1.2 ± 0.2				1.4 ± 0.4			
Rup AN	1.3 ± 0.3	=0.06	0.35	2.08	1.3 ± 0.4	=0.24	0.59	1.28
Unrup AN	1.2 ± 0.2				1.4 ± 0.4			

Note: Rup AN, Unrup AN and AUC area indicates ruptured aneurysm, unruptured aneurysm, and area under receiver operating characteristic (ROC) curve, respectively. *P* < 0.05 represents significant difference in ROC analysis between two groups. ROC analysis was performed between normal and aneurysmal groups (including the three categories of total, unruptured and ruptured aneurysmal cases) in the MCA, BA, ICA and ACA bifurcations, respectively. MCA, middle cerebral artery; BA, basilar artery; ICA, internal carotid artery; ACA, anterior cerebral artery.

### LA and DA ratios in BA bifurcations

In BA bifurcations, the two lateral angles were significantly smaller (*P* < 0.01) but the angle φ1 was significantly greater (*P* < 0.001) in all aneurysmal bifurcations than in the normal bifurcations. No significant (*P* > 0.05) difference was observed in the diameter of bifurcating arteries between normal and aneurysmal cases (Table 2).

The LA and DA ratios were 1.2 ± 0.2 and 1.3 ± 0.4, respectively, for normal BA bifurcations and 1.3 ± 0.3 and 1.3 ± 0.2, respectively, for the total aneurysmal cases. A significant difference (*P* = 0.0004) existed in the LA ratio between normal and aneurysmal bifurcations. On the contrary, no statistically significant difference (*P* = 0.52) existed in the DA ratio between normal and aneurysmal cases. A significant difference existed in the LA ratio between normal and unruptured or ruptured aneurysmal cases (*P* = 0.0013 and *P* = 0.04, respectively). However, no statistically significant difference (*P* = 0.38) existed in the LA ratio between unruptured and ruptured cases (Fig. 3).

ROC analysis showed a significant difference in the LA ratio between normal and total aneurysmal or unruptured aneurysmal bifurcations (*P* = 0.001 and *P* = 0.002, respectively), however, no significant difference between normal and ruptured cases was observed (*P* = 0.19) (Table 3).

### LA and DA ratios in ICA bifurcations

In ICA bifurcations, the two lateral angles at bifurcations with ruptured aneurysms were significantly smaller (*P* < 0.05), whereas the diameters of parent and both daughter branches with ruptured aneurysms were significantly larger (*P* < 0.001) than in the normal bifurcations (Table 2).

**Figure 4:**
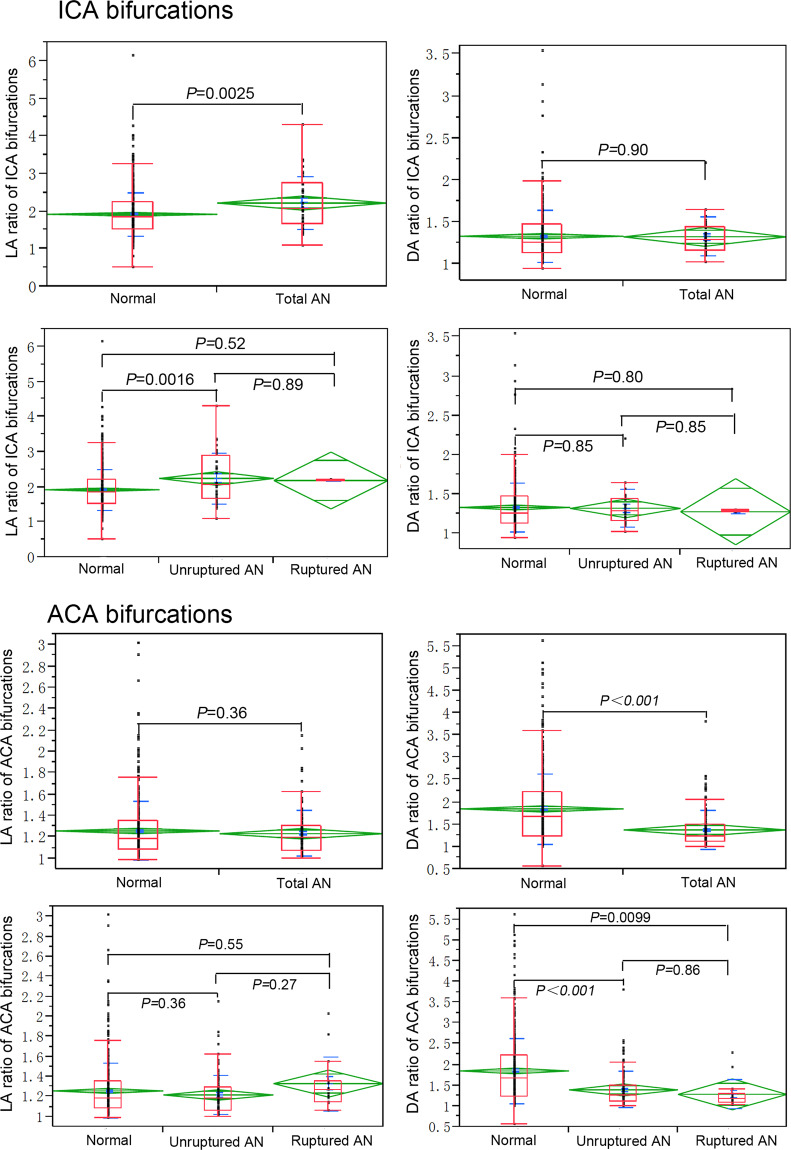
Comparison of LA and DA ratios in the internal carotid artery (ICA) and anterior cerebral artery (ACA) bifurcations between non-aneurysmal, unruptured and ruptured aneurysmal cases. Normal, control subjects without aneurysms; AN, aneurysms.

The LA ratio was significantly (*P* < *0.01*) smaller in the normal ICA bifurcations (1.9 ± 0.6) than in the total aneurysmal cases (2.2 ± 0.7) or in the unruptured aneurysm cases (2.2 ± 0.7) (*P* = 0.0016) (Fig. 4). However, no significant difference (*P* > 0.05) was observed in ROC analysis (Table 3). Similar to the BA bifurcations, no significant difference existed in the DA ratio between normal and aneurysmal cases (*P* > 0.05) (Fig. 4).

### LA and DA ratios in ACA bifurcations

The larger and smaller lateral angles and the diameter of bilateral daughter branches and A1 were all significantly smaller (*P* < 0.05), whereas the angle φ1 was significantly larger in aneurysmal ACA bifurcations (*P* < 0.001) than in those normal bifurcations (Table 2).

Different from the MCA bifurcations, the DA ratios in the total, unruptured and ruptured aneurysmal cases were all significantly smaller at the ACA bifurcations than in the normal cases (*P* < 0.001 and *P* = 0.0099, respectively). However, no significant difference existed in the LA ratio between normal and aneurysmal cases (*P* > 0.05) (Fig. 4).

The DA ratio was significantly (*P* < 0.01) different in the normal compared with the aneurysmal ACA bifurcations (including the three categories of total, unruptured and ruptured aneurysmal cases) in ROC analysis, however, no significant difference existed in the LA ratio in ACA bifurcations between the normal and aneurysmal bifurcations in ROC analysis (*P* > 0.05) (Table 3).

## Discussion

It has been reported that hemodynamic stresses play an important role in aneurysm initiation, formation and rupture^13^, and differences in vessel radii and asymmetric branch angles influence WSS magnitude and spatial distribution^14^. We thus hypothesized that morphological and hemodynamics differences may be associated with aneurysm presence. It has also been reported that aneurysmal bifurcations are associated with wider bifurcation angles^8,9^, however, whether or not significant differences exist in the lateral angles and diameters of daughter branches between normal and aneurysmal major cerebral arterial bifurcations remain to be established. In our present study, we examined the lateral angle (LA) ratio and daughter branch (DA) ratio at major cerebral MCA, BA, ICA and ACA bifurcations to determine the morphological parameters that are associated with cerebral aneurysms presence.

Our previous studies revealed that ACA and BA aneurysmal bifurcations had wider bifurcations and smaller lateral angles compared with normal cases^8,9^, however, the association between two lateral angles and aneurysm presence in major cerebral bifurcations is not clear. Our previous research also showed that ACA and BA bifurcation aneurysms mainly deviated to smaller lateral angles and smaller daughter branches^8,9^, inferring that the smaller daughter artery forming a smaller angle with the parent vessel may have abnormal hemodynamic stresses leading to vessel wall reconstruction. Sadatomo *et al*.^10^ have reported a significant difference in the LA ratio (the lateral angle formed between M1 and the larger M2 /the lateral angle between M1 and the smaller M2) between normal and aneurysmal MCA bifurcations. In our present study, the LA ratio was 1.8 ± 0.7 in the MCA bifurcations with aneurysms, significantly greater (*P* < 0.001) than those without aneurysms (1.3 ± 0.3), which is in agreement with the previous study by Sadatomo *et al*.^10^. This implies that aneurysmal MCA bifurcations are asymmetrical in the lateral angles (Fig. 3). Moreover, the LA ratio was 1.3 ± 0.3 and 2.2 ± 0.7, respectively, in the BA and ICA bifurcations with aneurysms, significantly larger (*P* < 0.01) than those without aneurysms (1.2 ± 0.2 and 1.9 ± 0.6, respectively) (Figs. 3, 4), which is also in line with the comparing analysis result of MCA bifurcations. It is interesting that the LA ratio in the ACA bifurcations harboring aneurysms showed a tendency to be lower than those of normal cases, even though there was no significant difference (*P* = 0.36) (Fig. 4).

The ROC analysis is commonly used when evaluating diagnostic tests. In our present study, significant differences (*P* < 0.01) existed in the LA ratio of normal MCA bifurcations compared with total aneurysmal, ruptured or unruptured aneurysmal bifurcations, with the AUC index of 0.74, 0.86 and 0.73, respectively, which is in agreement with previous study^10^. This may indicate that a greater LA ratio represents a higher risk of aneurysm presence at the MCA bifurcations. We hypothesized that the use of one stent deployed endovascularly through smaller lateral angle clinically could efficiently widen the smaller lateral angle and reduce the LA ratio, consequently improving the symmetry of the arterial bifurcation and decreasing the hemodynamic stresses and risk of aneurysm recurrence. A significant (*P* < 0.01) difference in the LA ratio was also observed between normal and BA bifurcations with aneurysms by ROC analysis. However, no significant difference existed in the LA ratio between normal and aneurysmal ICA or ACA bifurcations by ROC analysis (*P* > 0.05) (Table 3).

Arterial diameter is another morphological parameter to affect bifurcation asymmetrical structure. In this study, the DA ratio was calculated and compared between normal and aneurysmal cases. As a result, the DA ratio in MCA aneurysmal cases was significant larger than in the normal ones (*P* < 0.05), which is in accordance with the previous report^10^. Because our previous studies had revealed that cerebral aneurysms mostly deviated to smaller daughter branches^8,9^, we hypothesized that smaller artery with thin arterial wall and abnormal hemodynamic stresses may be associated with aneurysm presence. Our further research will focus on hemodynamic stress differences between bilateral daughter branches at normal and aneurysmal bifurcations to evaluate the relationship between asymmetric structure and hemodynamic stresses for aneurysm presence, and a possible link may be established between abnormal hemodynamic stresses and aneurysm initiation so as to better understand the morphological factors affecting presence of aneurysms. In our current study, no significant difference (*P* > 0.05) existed in the DA ratio between normal and BA or ICA aneurysmal cases (Figs. 3, 4), inferring that it is the lateral angle asymmetry rather than the daughter branch diameter that may play a more important role in aneurysm presence at BA and ICA bifurcations. Interestingly, we found for the first time that the DA ratio in ACA bifurcations with aneurysms was significantly smaller than in normal ACA bifurcations (*P* < 0.01) (Fig. 4), inferring that the ACA bifurcation harboring an aneurysm was relatively symmetrical when compared with normal cases without aneurysms, which was different from other arterial bifurcations. It has been reported that the anterior communicating artery (ACoA) aneurysm is more likely to be present at an ACA bifurcation with asymmetrical bilateral A1 segments^15^, however, the association between ACoA complex morphology and aneurysm presence remains unclear. ACoA complex is a very intricate anatomical region, which is composed of A1 and A2 segments of ACA and ACoA itself. The ACoA is an important communicating artery which links the bilateral anterior cerebral arteries. In normal physiology condition with a well-developed Willis circle, the ACoA does not normally participate in supplying blood to the brain. The ACoA will become a functional artery when unilateral dysplasia or aplasia of the A1 segment is present or when distal ICA is occluded^16^. Functional absence of ACoA in well-developed Willis circle may possibly present an asymmetrical geometry structure in normal ACoA complex.

The ROC analysis of the DA ratio showed that the DA ratio in normal ACA bifurcations was significantly (*P* < 0.001) greater than in ACA bifurcations (1.8 ± 0.8 vs. 1.4 ± 0.4) with aneurysms, with the AUC index of 0.70. Moreover, it was revealed for the first time that the DA ratio in ACA bifurcations with aneurysms, regardless of the ruptured status of aneurysms, tend to be relatively symmetrical when compared with normal subjects with no aneurysms. The DA ratio was significantly (*P* < 0.001) smaller in normal MCA bifurcations than in the total aneurysmal and unruptured aneurysmal MCA bifurcations, however, no significant difference (*P* > 0.05) was observed between normal and ruptured cases by ROC analysis, which was consistent with that of a previous report^10^.

With fast technical advancement of three-dimensional angiography, an increasing number of unruptured aneurysms can be detected. Although the rupture rate of aneurysms is only 1%, the consequence of rupture is severe with high morbidity and mortality rates^17^. Clinically, a decision to treat an incidentally-found unruptured aneurysm must be based on an effective prediction of the rupture possibility of the aneurysm. It has been reported that aneurysm size, location and aspect ratio (aneurysm depth/aneurysm neck width, and maximum dimension of the dome/aneurysm neck width) all are risk factors for rupture^18–21^, which are all associated with aneurysm morphology. Sadatomo *et al*.^10,11^ have shown that the DA ratio rather than the LA ratio in ruptured MCA bifurcation aneurysm cases was significantly smaller than the unruptured ones by *t* test and ROC analysis. However, no significant difference (*P* > 0.05) existed in the LA and DA ratio in the MCA, BA, ICA and ACA bifurcations between ruptured and unruptured aneurysmal cases in the present study.

Because asymmetrical cerebral arterial bifurcations are associated with aneurysm presence, deployment of one or two stents at bifurcations for assisting coil embolization of bifurcation aneurysms may decrease the bifurcation angle but increase the stented lateral angles, consequently altering the bifurcation geometry. Gao *et al*.^22–24^ have studied alteration of arterial bifurcation angles and lateral angles following stent deployment in the bifurcation for assisting coil embolization of bifurcation aneurysms and found that single or double stenting in Y configuration can significantly increase the stented lateral angle but decrease the bifurcation angle, displacing and attenuating the flow impingement zone and hemodynamic stresses (wall shear stress and total pressure) at the bifurcation apex, which may affect aneurysm initiation, development and evolution. This may indicate that stent-assisted coiling of bifurcation aneurysms can be considered when treating an intracranial bifurcation aneurysm in order to reduce risk of recurrence.

This study may have some limitations. One is that no analysis on hemodynamic stresses caused by asymmetrical or symmetrical morphological bifurcations was performed in this study. Hemodynamic stresses play an important role in aneurysm initiation, development and rupture, and changes of bifurcation angles and morphology are associated with significant hemodynamic stress alteration to affect aneurysm initiation and development. Our next step is to analyze the hemodynamic stresses in asymmetrical and symmetrical morphological bifurcations affecting aneurysm initiation and development. Another limitation may be a limited number of patients who were enrolled in this study, and future studies will have to recruit more patients for better outcomes. This study enrolled only Chinese people, and the results may be applied only to the Chinese ethnicity. Future studies will have to enroll people of multiple ethnicities.

In summary, normal MCA, BA and ICA bifurcations show a close-to-symmetrical morphology with smaller bifurcation angles but greater lateral angles, whereas aneurysmal bifurcations demonstrate an asymmetrical morphology in lateral angles and branch diameters. Normal ACA bifurcations show an asymmetrical morphology while symmetrical branches are associated with ACA bifurcation aneurysm presence.
