# Editorial Expression of Concern: Asymmetrical than symmetrical cerebral arterial bifurcations are more vulnerable to aneurysm presence

**DOI:** 10.1038/s41598-022-05082-5

**Published:** 2022-01-14

**Authors:** Xue-Jing Zhang, Wei-Li Hao, Dong-Hai Zhang, Bu-Lang Gao

**Affiliations:** 1 Department of Medical Research, Shijiazhuang First Hospital, Shijiazhuang, China; 2 Henan Balance Medial Laboratory, Henan Balance Medical Company, Zhengzhou, China

Editorial Expression of Concern to: *Scientific Reports* 10.1038/s41598-019-53715-z, published online 20 November 2019

The Editors are issuing an Editorial Expression of Concern for this Article because a concern has been raised that the imaging data presented originate from the patient database of a different institution from that described in the article. The authors have provided their raw data; however, the Editors have not been able to verify the provenance of these data with the authors' institution. The Editors therefore advise readers to interpret the data presented with caution. Xue-Jing Zhang, Wei-Li Hao, Dong-Hai Zhang and Bu-Lang Gao disagree with this statement.

